# The impact of scheduling ketamine as an internationally controlled substance on anaesthesia care in Sub-Saharan Africa: a case study and key informant interviews

**DOI:** 10.1186/s12913-024-11040-w

**Published:** 2024-05-07

**Authors:** Gaby I. Ooms, Mohammed A. Usman, Tim Reed, Hendrika A. van den Ham, Aukje K. Mantel-Teeuwisse

**Affiliations:** 1 Health Action International, Overtoom 60-2, 1054HK Amsterdam, The Netherlands; 2 Utrecht WHO Collaborating Centre for Pharmaceutical Policy and Regulation, Division of Pharmacoepidemiology and Clinical Pharmacology, Utrecht Institute for Pharmaceutical Sciences (UIPS), Utrecht University, Utrecht, The Netherlands; 3 Federal University of Dutse, Dutse, Jigawa State Nigeria; 4 Rasheed Shekoni Teaching Hospital, Dutse, Jigawa State Nigeria

**Keywords:** Ketamine, Anaesthesia care, Access, Drug policy, Sub-saharan Africa

## Abstract

**Background:**

Access to anaesthesia and surgical care is a major problem for people living in Sub-Saharan Africa. In this region, ketamine is critical for the provision of anaesthesia care. However, efforts to control ketamine internationally as a controlled substance may significantly impact its accessibility. This research therefore aims to estimate the importance of ketamine for anaesthesia and surgical care in Sub-Saharan Africa and assess the potential impact on access to ketamine if it were to be scheduled.

**Methods:**

This research is a mixed-methods study, comprising of a cross-sectional survey at the hospital level in Rwanda, and key informant interviews with experts on anaesthesia care in Sub-Saharan Africa. Data on availability of four anaesthetic agents were collected from hospitals (*n* = 54) in Rwanda. Semi-structured interviews with 10 key informants were conducted, collecting information on the importance of ketamine, the potential impact of scheduling ketamine internationally, and opinions on misuse of ketamine. Interviews were transcribed verbatim and analysed using a thematic analysis approach.

**Results:**

The survey conducted in Rwanda found that availability of ketamine and propofol was comparable at around 80%, while thiopental and inhalational agents were available at only about half of the hospitals. Significant barriers impeding access to anaesthesia care were identified, including a general lack of attention given to the specialty by governments, a shortage of anaesthesiologists and migration of trained anaesthesiologists, and a scarcity of medicines and equipment. Ketamine was described as critical for the provision of anaesthesia care as a consequence of these barriers. Misuse of ketamine was not believed to be an issue by the informants.

**Conclusion:**

Ketamine is critical for the provision of anaesthesia care in Sub-Saharan Africa, and its scheduling would have a significantly negative impact on its availability for anaesthesia care.

**Supplementary Information:**

The online version contains supplementary material available at 10.1186/s12913-024-11040-w.

## Introduction

Surgical care is defined by the Lancet Commission on Global Surgery as “the provision of operative, perioperative, and non-operative management; anaesthesia; and obstetric care for all surgical conditions” [1]. Surgical care is a cross-cutting field of care, and surgical procedures are essential in the treatment of communicable and non-communicable diseases, maternal, neonatal and nutritional disorders, and injuries [1]. It is estimated that conditions requiring surgery are responsible for around 30% of the global burden of disease, while access to safe, affordable and timely surgical and anaesthesia care is a major issue for more than 4.8 billion people worldwide [2, 3]. This treatment gap is felt the most by people living in low- and middle-income countries (LMICs): an additional 143 million surgical procedures are needed in LMICs annually to avert preventable disability and deaths, and more than 77 million disability-adjusted life-years (DALYs) could be averted with adequate provision of basic surgical care [1]. Anaesthesia is a key component of surgical care.

Access to to timely, safe and affordable surgical and anaesthesia care is a major problem for people living in Sub-Saharan Africa (SSA), where it is beyond the reach of more than 95% of the population [3]. Lack of access to surgical and anaesthesia care in SSA is caused by a paucity of specialised healthcare workers, poor basic infrastructure, absence of surgical and anaesthesia equipment, and scarcity of essential medicines, including anaesthetic agents [4]. It is estimated that in the World Health Organization (WHO) Africa Region, there are on average 0.41 physician anaesthesia providers (PAPs) per 100,000 population. This number is far below the 10 PAPs per 100,000 population as recommended by the World Federation of Societies for Anaesthesiologists (WFSA) [5]. Research has shown that consistent access to electricity and running water remains problematic across SSA, and that availability of oxygen and functional anaesthetic machines is generally low [6–18]. Essential medicines, such as local or general anaesthetic agents, remain in low supply [6, 9, 17, 19–21].

Due to the lack of PAPs, infrastructure, equipment, and essential medicines in much of SSA, surgical procedures often take place without adequate anaesthesia or pain management [22]. To alleviate the suffering of patients in these settings, hospitals have become reliant on ketamine. The WHO Model List of Essential Medicines lists ketamine for use as an anaesthetic [23, 24]. Its use in low-resource settings is popular as ketamine does not depress respiratory function in patients while it increases blood pressure, and can therefore be used when access to airway equipment is lacking and monitoring of vital signs is challenging [22, 23]. Because of these properties, ketamine can also be used by non-physician providers, if they have been appropriately trained [22].

Ketamine is misused as a recreational drug in high-income countries, especially in China, Hong Kong, Taiwan, and Japan, and more generally in East and Southeast Asia [4, 23, 25]. Because of this misuse, China has repeatedly submitted a request to schedule ketamine as a Schedule I drug under the Single Convention on Narcotic Drugs in 2006, 2012 and 2014, and submitted a request to have it scheduled as a schedule IV drug under the Convention on Psychotropic Substances in 2015 [23, 26–29], see Box 1 for detailed information.

**Box 1:** Scheduling of medicines under the Conventions

Drugs scheduled as a Schedule I drug in the Single Convention are subject to all measures of control under the Convention; only Schedule IV drugs are more tightly regulated within this Convention [28]. Measures include, amongst others, obligatory annual estimates, full documentation of quantities produced, manufactured, used, imported and/or exported, and special licenses for distribution.
Schedule IV substances in the Convention on Psychotropic Substances are subject to control measures such as special licenses for manufacture, trade and distribution, full documentation similar to the Single Convention provision, import and export is only allowed when tightly regulated, and countries are allowed to prohibit the import of any psychotropic substance [29].

All four instances that scheduling was requested, the WHO Expert Committee on Drug Dependence (ECDD) declined, stating that ketamine poses no great global public health risk, while scheduling it would have a significant impact on medical care in LMICs and in emergency situations [25–27]. Subsequently, the United Nations Commission on Narcotic Drugs (CND) has not scheduled ketamine as a Schedule I drug in the Single Convention, or as a Schedule IV drug in the Convention on Psychotropic Substances [25–27]. However, it is likely that similar requests will be made in the future.

While the importance of ketamine for anaesthesia care has been discussed in an article in the Guardian and in editorials, no research has been undertaken in which anaesthesiologists from the field provide their insights into the issue [26, 27, 30, 31]. This research therefore aims to estimate the importance of ketamine for anaesthesia and surgical care in SSA, and assess the potential impact on access to ketamine if it were to be scheduled, through a case study of essential anaesthesia commodities availability in Rwanda, and key informant interviews with experts from SSA.

## Methods

### Study design

This research is a mixed-methods study, comprising of a cross-sectional survey at the hospital level in Rwanda, and key informant interviews with experts on anaesthesia care in SSA. The survey on the availability of anaesthesia commodities was part of a larger project in Rwanda on access to essential medicines for the management and treatment of snakebites [32]. In this study, 34 commodities were surveyed, including four commodities that are used in anaesthesia care (ketamine, thiopental, inhalational agents, and propofol). The survey functioned as a case-study to gain insight into the availability of a range of anaesthesia commodities in a specific SSA country. Semi-structured interviews were conducted with key informants from the whole of SSA to gather a more generalised insight into the importance of ketamine for anaesthesia and surgical care in the entire region, given the situation in Rwanda may not be representative of the region.

### Study participants and recruitment

In Rwanda, all general, non-specialised hospitals from the public and private sectors were selected for the survey. This included four private hospitals, and 51 public district-, provincial- and referral hospitals. The hospitals were contacted beforehand by email or telephone to schedule a study visit. One specialised hospital solely focussing on psychiatric care was excluded as were lower level health facilities, including health centres and health posts. These facilities were not included since they were not expected to have (most) anaesthetic agents.

Key stakeholders identified for participation in the interview component of this study were anaesthesiologists with expertise in anaesthesia care in SSA. They were identified and recruited through document desk review, the network of the WFSA and its national chapters, and the professional network of the researchers. Inclusion criteria for participation were: participants are 18 years or older, knowledgeable on anaesthesia care and ketamine use in SSA, and able to communicate in English. Participants were invited over email and provided with background information on the study. Multiple follow-up emails were sent in case of non-response.

### Data collection

The WHO-WFSA *International Standards for a Safe Practice of Anesthesia* guided the selection of the general anaesthesia commodities [33]. Information on electricity, running water, and functional anaesthesia machines was also recorded. Data within the Rwandan hospitals was collected in February 2023. A mobile application, KoboCollect, was used for data collection. Data collectors received a two-day training from one of the authors (GIO), which included a field-test. Data collectors collected data in pairs and were supervised by an in-country lead investigator. Data on availability of the commodities was recorded only when they could be physically seen. A commodity was considered available if it was present at the hospital at the time of data collection. A photo was taken of each available, surveyed commodity as an additional validity measure.

A semi-structured key-informant interview guide was developed based on literature to guide the interviews (see Additional File 1). Questions focused on the contextual situation of anaesthesia care, including barriers to access, in the countries in which participants have work experience, their beliefs about ketamine and its relevance for anaesthesia care in these respective countries, and their perceived potential impact of ketamine scheduling on anaesthesia and surgical care in these contexts. We also sought the participants’ opinions on the level of misuse of ketamine in their countries, and about recommendations to safeguard access to anaesthesia care while at the same time preventing misuse of ketamine. Interviews were conducted by GIO from May to July 2023 with 10 participants. Nine interviews took place online through virtual meeting platform Zoom, and one interview was conducted via email, where the key informant responded to the questions in written form due to language barriers. Interviews lasted between 27 and 53 min. Interviews were recorded, and Zoom’s build-in automatic transcription setting was used.

### Data management and analysis

Survey data were uploaded to the KoboToolbox server by the data collectors after completion, after which the data was downloaded into Microsoft Excel. The data was double-checked and cleaned by the researchers, and was analysed in Microsoft Excel using descriptive statistics. Availability across all hospitals was calculated as the proportion of hospitals where the commodity was present at the time of the survey.

The automatic, verbatim interview transcripts were checked by the researchers for errors and corrected when necessary after a consensus was reached. The interviews were analysed using a thematic analysis approach by one researcher (GIO), and consisted of coding text into predetermined themes, which were based on the interview topics.

### Quality assurance

The qualitative component of this research was guided by the Consolidated Criteria for Reporting Qualitative Research (COREQ) framework (see Additional File 2) [34]. Triangulation occurred in two ways: informant triangulation through the inclusion of stakeholders from multiple countries, and data triangulation through the use of both quantitative and qualitative research methods. Transferability of the research is increased through a detailed description of the context of the research, the data collection, and data analysis.

### Ethical considerations

In accordance with the Declaration of Helsinki, ethical approval for the hospital survey was granted by the University of Global Health Equity Institutional Review Board, approval number UGHE-IRB/2022/056, and by the Rwanda National Health Research Committee, approval number NHRC/2022/PROT/050. Ethical approval for the interviews was granted by the Ethics Review Board of the faculties of Science and Geosciences, Utrecht University, approval number S-23,008. Informed consent was given by all participants (see Additional File 3).

## Results

### Availability of anaesthetic commodities in Rwandan hospitals

In total, 54 hospitals participated in this study. One hospital declined participation. The general anaesthetic with highest availability was propofol (81.5%), followed by ketamine (77.8%). Inhalational agents, such as halothane, isoflurane or sevoflurane were available at 53.7% of the hospitals, and thiopental at 44.4%. All hospitals had running water and electricity, and 90.7% had a functional anaesthetic machine.

**Fig. 1:**
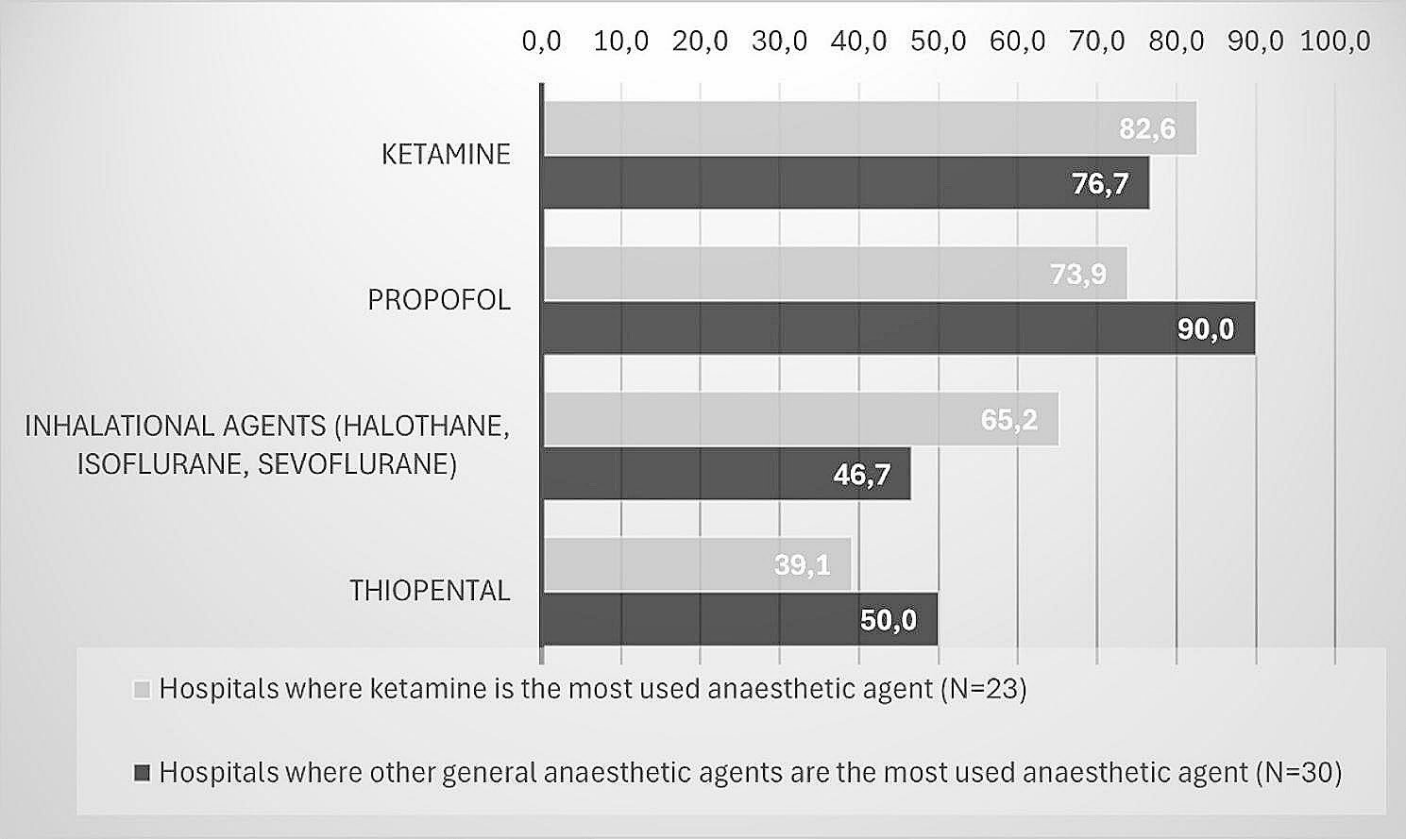
Anaesthetic agents’ availability in hospitals, stratified by self-reported most used anaesthetic agent

Ketamine was indicated as the general anaesthetic agent most used by 23 hospitals (42.6%). Twenty hospitals (37.0%) indicated it was propofol, while the remaining ten medical professionals (18.5%) indicated it was halothane. Data was missing for one hospital. In the hospitals where ketamine was the most used anaesthetic agent, it was also the anaesthetic agent with the highest availability at 82.6% (see Fig. 1). In the 30 hospitals where other general anaesthetic agents were indicated to be most used, highest availability was found for propofol (90.0%, see Fig. 1).

### Key informant interviews

Sixty-nine individuals or national anaesthesia societies were contacted for participation in the study, of which ten agreed to participate. Key informant characteristics are provided in Table 1. Nine informants were knowledgeable about a country-specific context, while one informant (a WFSA member from Europe) had knowledge about the Sub-Saharan region in general.

**Table 1:** Key Informant characteristics

Participant number	Country / region	Profession	Sex
P1	Europe	Anaesthesiologist	Female
P2	Democratic Republic of Congo	Anaesthesiologist	Male
P3	Ethiopia	Anaesthesiologist	Male
P4	The Gambia	Anaesthesiologist	Male
P5	Namibia	Anaesthesiologist; critical care	Male
P6	Nigeria	Anaesthesiologist	Male
P7	Somaliland	Nurse-anaesthetist	Male
P8	South Africa	Anaesthesiologist	Female
P9	Zambia	Anaesthesiologist; critical care	Male
P10	Zimbabwe	Anaesthesiologist	Female

### Barriers to anaesthesia care

Multiple barriers to anaesthesia care were highlighted by the key informants. One of the main issues raised by all participants, was the lack of anaesthesiologists (Table 2, Quote 1). The number of anaesthesiologists was said to be critically low, with all anaesthesiologists primarily located in urban locations, in the more specialised hospitals. The key informant from the Democratic Republic of Congo (DRC) sketched this situation (Table 2, Quote 2). As a consequence, anaesthesia care is provided by non-physician providers, such as nurses and medical officers. However, eight of the key informants reported that these non-physician providers often had limited training in anaesthesia care, and do not have the skills or knowledge to provide more complex anaesthesia. This issue was highlighted by the key informant from Zambia (Table 2, Quote 3). One key informant also reported that protocols are not followed in some locations when providing anaesthesia care.

**Table 2:** Barriers to accessing anaesthesia care, selected quotes

Quote number	Quote (participant number)
1	*“In Somaliland still, they don’t have any single local physician anaesthesia provider.”* (P7)
2	“*The DRC is a large country with more than one hundred million inhabitants, but the number of anaesthetists is still low, less than 100 and all concentrated in the big cities: Kinshasa the majority, Lubumbashi (5 and doctors in training), East of the country (six), Central Kongo (two), and the rest of the provinces do not have anaesthetists and therefore the anaesthesia is done by anaesthesia technicians (anaesthesia nurses) or even nurses and general practitioners.*” (P2)
3	“*Very, very few of the of the hospitals in Zambia have physician anaesthesiologists. Most of them have people that are below that level of training, and so they may not be able to provide very complex anaesthetics.*” (P10)
4	“*More than 85% of our anaesthetists in South Africa that qualifies annually, leaves for the private sector. And the private sector sees less than 40% of the patient burden. So the number is really very skewed in our country. And now, with all the economic things that is happening, a lot of us are leaving the country as well.”* (P8)
5	“*In general, anaesthesia care is growing. But it is highly challenged by availability of equipment and drugs. Like modern equipment, anaesthesia machines, monitoring equipment, like in the ICU too, […] and drugs like sevoflurane, the wide variety of modern drugs are lacking, it’s not available. Access is highly limited.*” (P3)
6	“*I would say the biggest barrier is maybe ignorance about the importance of anaesthesia. What anaesthesia’s role is in the hospitals, and how big of an impact a good anaesthetic service would have on our health system. I think that ignorance translates into poor funding into the field. It translates into poor recruitment. It translates into poor sponsorships for healthcare workers who do want to study anaesthesia.*” (P9)

DRC: Democratic Republic of Congo; ICU: Intensive Care Unit

Another issue raised by the two informants from South Africa and Zimbabwe, was migration of trained anaesthesiologists, both within the country and abroad. For example, anaesthesiologists moved towards the private sectors, as they are offered better wages and working conditions there (Table 2, Quote 4). Crucially, the lack of medicines and equipment was also a significant barrier to anaesthesia care. Nine of the informants reported that the lack of medicines and equipment experienced in health facilities impedes the provision of anaesthesia care (Table 2, Quote 5). The respondent from Zimbabwe mentioned that the government, as part of the National Surgical, Obstetrician and Anaesthesia Strategy is purchasing equipment to tackle this problem. In South Africa, the respondent shared that availability of medicines has improved and is not a major issue there.

Lastly, four informants specifically mentioned the lack of training opportunities and attention, and subsequently the lack of budget, given to anaesthesia care. The informant from Ethiopia referred to the government’s primary policy focus on prevention of infectious diseases, not on chronic diseases. The respondent from Namibia shared that only since 2018, doctors can train to become anaesthesiologists as part of the Namibian medical curriculum; before they needed to travel to other countries, such as South Africa, to study. In the Gambia there is no training available yet for anaesthesiologists. The informant from Zambia referred to the lack of attention among medical professionals and the public, as well as policy makers, as the main barrier to anaesthesia care (Table 2, Quote 6).

### Ketamine for anaesthesia care

Ketamine was described as critical for the provision of anaesthesia care in their respective countries by all of the key informants. Five of the informants reported that in more specialised hospitals, where anaesthesiologists provide anaesthesia care, propofol, also a non-controlled substance, was the preferred anaesthetic. However, ketamine is also commonly used in these hospitals, specifically for haemodynamically unstable patients, hypotensive patients, patients who are in shock, and as a sedative in paediatric patients, patients with asthma or patients on the intensive care unit (ICU). Ketamine is also used for pain management. Four of the informants also referred to shortages of anaesthetic agents, such as propofol, that occurred in the specialised hospitals, which made them reliant on ketamine (Table 3, Quote 1).

**Table 3:** The importance of ketamine for anaesthesia care, selected quotes

Quote number	Quote (participant number)
1	“*Propofol is the preferred one. The issue is, it’s costly, and its availability is limited. […] So I would say, until recently, the majority of the cases are being induced by ketamine. But you know, request for propofol is highly increasing. We are getting, at least at my institution, We are getting more propofol these days.*” (P3)
2	“*Ketamine is very important in DRC because it is available, cheaper, and easy to use even without an anaesthesia machine. Everyone – specialists, general practitioners, nurses – can use it.*” (P2)
3	“*Some […] of the providers don’t have the skill or knowledge of how to perform a spinal anaesthetic, and the majority of surgeries that are done in rural settings tend to be for obstetric emergencies in which a spinal anaesthetic may be, would be warranted. But because they don’t have that skill, they would prefer to use a drug like ketamine that would […] keep the patient breathing on their own, and would allow for surgery to be done.*” (P9)
4	“*Because out of the operation theatre, the [health] facility, in case if the patient lost breathing effort, the facility is not appropriate. So we will feel safe only when we are using ketamine, because, as compared to other sedative agents, its adverse effects, loss of breathing and so on, is very much minimal with ketamine compared to others. Because of all this, I think I would say ketamine is very important, you know.*” (P3)
5	*“Ketamine is about the cheapest. The one we have here […] so that bottle is sold, in our local currency, that’s about 500 Naira which is less than 1 USD. So, yeah, so it’s always available. Propofol goes for 2,500 per ampoule. And that is about 4 times or 5 times the price of ketamine. Now, fentanyl goes for about 5,000 Naira. Which is about 10 times the price of ketamine. […] Then for regional anaesthesia, we’re using bupivacaine, bupivacaine goes for 4,000. Which is about 8 times the price of ketamine per ampoule. So ketamine is somehow cheap and is available for us to use.*” (P6)
6	“*Ketamine, eight months ago I would have said that number is very close to 100%. Because of how important it was. But with what’s happening right now, the supply, I would say maybe under 10% of hospitals have it. We are one of the largest hospitals in the country, and we don’t have ketamine. And usually we’re the last to get hit. So I think that if we don’t have ketamine I can’t imagine many others will.*” (P9)

DRC: Democratic Republic of Congo; ICU: Intensive Care Unit

One of the primary reasons given for the importance of ketamine by all of the informants, is that it can easily be used by non-physician providers, who provide the bulk of anaesthesia services, especially in rural areas (Table 3, Quote 2). Informants shared that non-physician providers prefer to use ketamine as they are uncomfortable providing anaesthesia with alternatives because of potential side effects. Further, these providers often have only received a basic training in anaesthesia care and are not conversant with providing other anaesthetic agents (Table 3, Quote 3). Related, in lower-level hospitals and in rural areas, a lack of equipment, such as anaesthetic machines, exacerbated the difficulties of providing anaesthesia, and increased the reliance on ketamine, as they were fearful of the adverse consequences, and the possibility of death, when using other anaesthetic agents (Table 3, Quote 4). The informants from Somaliland and Nigeria raised the issue of affordability of medicines, and that next to ketamine being the most available anaesthetic agent, it was also the most affordable (Table 3, Quote 5).

When the key informants were asked about the availability of anaesthetic agents in their respective countries as compared to the findings of the survey conducted in Rwanda, variations were reported. First, the informants emphasised it is difficult to report exact availabilities of the anaesthetic agents without conducting a similar survey. However, the informants shared that ketamine availability would be similar, or even higher, in their countries. Zambia was an exception, as the informant reported that for months preceding the interview, there had been critical shortages of ketamine (Table 3, Quote 6). The informant did not know the reason for the shortages. Informants from the DRC, Ethiopia, Nigeria, Somaliland and Zambia shared that the availability of propofol would be (slightly) lower than in Rwanda, especially in rural hospitals, while the informants from the Gambia, Namibia and South Africa shared that it would be more or less similar.

### Misuse of ketamine

None of the key informants reported that misuse of ketamine was a significant issue in their respective countries, as far as they were aware. Three informants offered anecdotal evidence of specific instances of misuse that they knew or had heard about, although the case reported by the informant from the DRC about a sickle cell patient misusing it for the treatment of vaso-occlusive crisis is strictly speaking not about misuse but unlicensed use. Two informants shared that there was some misuse of ketamine among medical professionals in their countries. The Zambian informant shared that a medical professional had died as a consequence of the misuse. The South African informant reported that while she was aware of medical professionals that had misused ketamine and this issue should not be overlooked, the balance between control and access should be kept in mind (Table 4, Quote 1).

**Table 4:** Ketamine misuse and international scheduling, selected quotes

Quote number	Quote (participant number)
1	“W*ith the Schedule 5 [of ketamine] in South Africa, ketamine is also still locked. And ketamine is also still signed for. And I think education and the enforcement of patient-by-patient administration and access to ketamine is the best way for patient care and for protecting the provider, from […] exposing themselves to the risk of ketamine misuse.*” (P8)
2	“*So when ketamine is being abused in other places, then it’s likely that it will come here later. So I mean restricting those drugs not to be accessible for individuals, other than hospitals, has to be, I think, considered. But now it is not a major of a concept.*” (P3)
3	“*In smaller hospitals, however, I have found that it’s not as tightly controlled. So the ketamine ampoule will be given, and it will be placed on your product trolley for the day.*” (P8)
4	“*I think in our local hospitals, there should be protocols on who to use ketamine. So if there are protocols and there are controls within the hospitals, such that whoever uses ketamine signs in and signs out. […] Whatever prescription has been, that he has written, should be stated clearly so that such can be traced. And also people handling ketamine. So we can now start using it as a [controlled] drug within the anaesthesia room. Such that it is not left in the open. So that it is only accessed when we need to use it.*” (P6)
5	“*I think it’s going to affect a lot of us who practice in rural communities. Because one, it’s going to affect the availability. And how we access. And it’s also going to make it very, very expensive. Because there will be a lot of controls, bottlenecks, trying to import ketamine, and make it available.”* (P6)
6	*“It will just affect it as it is affecting the opioid supplies in our country. And having an opioid medication for analgesia is the hardest challenge that one can have. And we know the exact reason why. Because of the categorization of the medication*.” (P4)
7	“*We can’t be seen as part of the international group, if our resources and operational profile is completely different. I mean, I don’t think it can be standardized that a drug that can potentially be life-saving, and a drug that is definitely part of our armoury for effective analgesia in a resource-limiting setting, that we are then under the same strict scheduling as a developed country that might have access to multiple other options.*” (P8)

In all the other countries, the informants were unaware of misuse cases among medical professionals. Additionally, all of the informants shared that ketamine misuse among the general public was not an issue. The informant from Nigeria shared their opinion that ketamine may be misused among the internally displaced. Some of the informants also shared that if misuse is occurring in high-income countries, it might eventually also happen in their countries (Table 4, Quote 2).

### International scheduling of ketamine as a controlled substance

In three of the nine countries in which the informants work, ketamine is scheduled or regulated to some extent at the national level. In the Gambia, Namibia and South Africa, ketamine is stored in a locked cabinet, and medical professionals are required to request ketamine, and the release is signed off in a logbook by both the requesting medical professional as well as an in-charge nurse. However, in Namibia and South Africa informants shared that this procedure is not always followed as tightly as it might need to be (Table 4, Quote 3). In the other countries, ketamine was not subject to additional, national control. Some of the informants from these countries could see the added value of having such controls at the national level for better stewardship (Table 4, Quote 4).

If ketamine were to be scheduled as a controlled substance at the international level, it was believed it would negatively impact access in the informants’ respective countries, especially in the more rural locations. They all emphasised the critical importance of ketamine (Table 4, Quote 5). Informants from Namibia, the Gambia and Somaliland also made the comparison to already controlled substances, fearing the availability of ketamine would decrease to similar levels (Table 4, Quote 6). Next to the availability, some informants also raised concerns about increased costs of ketamine as a consequence of its scheduling, which would hamper access. In Zambia, where there is currently a shortage of ketamine, the informant shared their fears of this being the new reality. Lastly, one of the informants argued that LMICs and high-income countries should not be subjected to the same measures as they have very different resources available to them (Table 4, Quote 7).

### Recommendations to improve access to anaesthesia care

Recommendations made by the key informants to improve access to anaesthesia care were related to increasing attention and budgets for anaesthesia care, training and retention of anaesthesiologists and non-physician providers, improving availability of medicines and equipment, and decentralisation of care. For example, the key informant from Europe representing the global view argued that countries need to take responsibility and put resources into anaesthesia care (Table 5, Quote 1). Similarly, the key informant from the Gambia argued for increasing the incentives to work in anaesthesia (Table 5, Quote 2). In line with this, the informant from Ethiopia argued for better collaboration between medical professionals and the Ministry of Health to ensure the medicines provided are the ones needed. The informant from Zimbabwe highlighted that, while much can still be improved, in the last few years, more and more attention has been paid to anaesthesia care. The informant from Nigeria pointed to COVID-19 for the increased availability of equipment, but also stressed the need for better policies without waiting for another pandemic to occur (Table 5, Quote 3). The importance of training of medical professionals was highlighted by the informant from Namibia (Table 5, Quote 4), while the informant from South Africa added the need to find a way to retain their trained specialists, as many are leaving to work in high-income countries. Last, the same informant also emphasised the importance of decentralisation of care, in which anaesthesiologists should go to rural areas to treat patients, instead of patients travelling far to come to the specialised hospitals in the big cities (Table 5, Quote 5).

**Table 5:** Recommendations to improve access to anaesthesia care, selected quotes

Quote number	Quote (participant number)
1	“*Unless the countries, governments, themselves do not take action, we will not succeed. So what has been done with those national anaesthesia, surgical and obstetric plans is important, that we must have the countries’ governments to take responsibility. And that goes for training, […] and all kind of medications we are using, and so on.*” (P1)
2	“*Incentivise the department of anaesthesia. Give more opportunity to those that are ready to go into it, because the competition is between specialities. So obviously everyone wants to go to an area where they have a better chance in their academic progress. So if you incentivise the department of anaesthesia, we will have so many clinicians or nurse anaesthetists who are giving safe anaesthetic care within the country*.” (P4)
3	“*There should be a policy, a deliberate policy by government. […] COVID-19 came with a lot of problems. it opened our eyes to our emptiness. So after COVID-19, a lot of things have been done, provided. For example, anaesthetic machines, monitors, multi-parameter monitors, and even pulse oximeters, and the rest of them. […] So what I will say is, we shouldn’t wait for such things to happen.*” (P6)
4	“*Training more people, having more staff in the department. So we have limited number of theatres, we’re trying to expand the number of theatres that we have, but one of the stumbling blocks is limited number of [staff in the] anaesthesia department. So we’re trying to push for more staff.*” (P5)
5	“*Decentralisation of care is definitely, I feel, a buzzword, and is something that we need to do nationally and in sub-Saharan Africa really look at. That we don’t spend all our money that is already limited, in bringing amounts and amounts of patients, 700, 900, 1000 km, them staying in hospital for three, four, five nights, versus two specialists travelling down, sleeping over and delivering the same quality of care at the patient. So I do think decentralisation is definitely the way to go in sub-Saharan Africa for us to make… to actually make our healthcare service accessible to our patients*” (P8).

## Discussion

This is a first-of-its kind research on the importance of ketamine as detailed by anaesthesiologists working in SSA. It also studied the availability of ketamine compared to other anaesthetic agents specifically in Rwandan hospitals. The interviews with the key informants from across SSA found that there were significant barriers impeding access to anaesthesia care, including a general lack of attention given to the speciality by governments, a shortage of anaesthesiologists and migration of trained anaesthesiologists, and a scarcity of medicines and equipment. Ketamine was described as critical for the provision of anaesthesia care as a consequence of these barriers, and its scheduling would have a significantly negative impact on the quality of anaesthesia care that can be provided. The survey conducted in Rwanda found that availability of ketamine and propofol was comparable at around 80%, while thiopental and inhalational agents such as halothane, isoflurane or sevoflurane were available at only about half of the hospitals.

These barriers to anaesthesia care identified in this study have been identified previously in different contexts, and this research supports those findings [4, 6–21]. When the key informants were asked whether the availability in their respective countries was comparable to the availability found in Rwanda, the responses were variable. This is in line with previous research studying the availability of anaesthetic agents [6, 17, 21]. For instance, a study from Liberia found that ketamine was available 76-100% of the time in 88% of surveyed facilities, and this was the case for propofol in only 46% of facilities [6]. Similarly, while anaesthesia using ketamine was available in 13 of 14 health facilities surveyed in Somalia, anaesthesia using inhalational agents was available at five of the facilities [17]. Further, all surveyed hospitals in Rwanda had running water and electricity. Previous studies in Nigeria and Somalia found that access to running water and electricity was not guaranteed; the study in Nigeria found that hospitals suffered daily power outages ranging from 10 to 22 h, and only 15% had running water [18]. In Somalia, 28% of surveyed health facilities never or only sometimes had access to running water, and only 50% had consistent access to electricity [17]. Last, in this study it was found that 90.7% of hospitals had a functional anaesthesia machine. In Tanzania, Nigeria and Somalia, 67%, 23% and 15% of hospitals, respectively, had a functional anaesthesia machine available [15, 17, 18].

The case study of Rwanda thus may not be representative of the availability in other countries in the region. However, this research has shown that even when other anaesthetic agents, such as propofol, are available, much of anaesthesia care is still provided using ketamine. This is due to the lack of trained anaesthesiologists, and the subsequent reliance on non-specialist anaesthesia providers, such as nurses and medical officers. These non-physician providers feel better prepared to provide anaesthesia using ketamine, as there are much fewer potential side-effects than the other agents. This has also been described elsewhere [27, 35]. Further, also in more specialised hospitals where anaesthesiologists are present to provide anaesthesia care, key informants shared ketamine is still one of the main anaesthetic agents used due to shortages of propofol that occur. A study conducted in district hospitals in Malawi, Zambia and Tanzania reported similar findings, showing that anaesthesia care at the district level is provided only by non-physician anaesthesia providers, and that ketamine was widely used to mitigate shortages of other anaesthetic agents [8].

In this study, the key informants reported that, as far as they were aware, misuse of ketamine is not a significant issue in their respective countries. A few did provide anecdotal evidence of specific instances of misuse among medical professionals. However, all informants believed scheduling ketamine internationally as a controlled substance would have a negative impact on access to anaesthesia care, as its availability would likely decrease. This fear is not unsubstantiated, as multiple informants referred to the difficulties with accessing opioids in their countries. In line with this, while in Liberia and Ethiopia ketamine was (almost) always available in 88% and 100% of facilities, respectively, morphine was (almost) always available at only 35% and 27% of facilities, respectively [6]. Consequences of international scheduling are restrictions on production, manufacturing, importation, distribution and use of medicines, resulting in severely limited access to controlled medicines [36]. It is thus paramount that ketamine does not become a scheduled substance. Instead, to safeguard against potential ketamine misuse in their respective countries, key informants believed in strengthening prescribing and dispensing practices in the healthcare setting. In many countries, ketamine is still freely available for all healthcare workers. Limiting ketamine so it is only obtainable for those allowed to use it may prevent future misuse. In Namibia, for example, ketamine is a Schedule 3 substance, and subsequently needs to be locked away and can only be sold or provided by designated personnel, on the basis of a prescription. The amount sold or provided has to be recorded in a logbook or prescription book [37].

### Limitations

While this is the first study collecting experts’ insights into the importance of ketamine for anaesthesia care in SSA, some limitations should be noted. In the survey conducted in Rwandan hospitals, no price or stock data was collected for the anaesthetic agents. This might have provided insights into the differences in costs between the different agents, and the availability over time. While the hospitals in Rwanda were contacted beforehand to schedule a visit for the survey, because the data collected for this study was part of a larger study on snakebites it is believed that hospitals could not have taken measures that might have changed the availability numbers. Further, while more than 60 individuals and national anaesthesia societies were contacted, only ten individuals agreed to participate. Of these, none were unfortunately from Rwanda and only two were from West Africa. Due to this low number of respondents, it is difficult to assess whether topical saturation was fully reached. However, after initial analysis of eight interviews, the subsequent analysis of the last two interviews did not yield new insights, indicating potential data saturation. This study thus gives a first, detailed insight into the importance of ketamine for anaesthesia care in SSA. Further research may be undertaken to tease out more detailed, contextual factors that may not have been caught in this study.

## Conclusion

This study has shown that ketamine is a critical medicine for the provision of anaesthesia care in SSA, as this field faces barriers related to its workforce and availability of medicines and equipment. If accessibility of ketamine changes as a result of its international scheduling, millions of people’s access to safe anaesthesia and surgical care will be in danger. Countries should strengthen prescribing and dispensing practices in the healthcare setting. Further, concerted efforts should focus on improving anaesthesia care in SSA in general, so in the future there can be less of a reliance on ketamine. Governments should focus more of their attention on the speciality, allocating more budget, facilitating training of more anaesthesiologists and non-physician providers, improving availability of medicines and equipment, as well as focusing efforts on retaining their anaesthesia workforce.

### Electronic supplementary material

Below is the link to the electronic supplementary material.

Supplementary Material 1

Supplementary Material 2

Supplementary Material 3

## Data Availability

The datasets used and/or analysed during the current study are available from the corresponding author on reasonable request.

## Abbreviations

CND: Commission on Narcotic Drugs
DALYs: Disability-adjusted life-years
DRC: Democratic Republic of Congo
ECDD: Expert Committee on Drug Dependence
ICU: Intensive care unit
LMICs: Low- and middle-income countries
PAPs: Physician anaesthesia providers
COREQ: Consolidated Criteria for Reporting Qualitative Research
SSA: Sub-Saharan Africa
WFSA: World Federation of Societies for Anaesthesiologists
WHO: World Health Organization
