# Supplementary material for: The impact of scheduling ketamine as an internationally controlled substance on anaesthesia care in Sub-Saharan Africa: a case study and key informant interviews
Source: BMC Health Serv Res. 2024 May 7;24:598. doi: 10.1186/s12913-024-11040-w (PMC11077710; doi:10.1186/s12913-024-11040-w)
Supplement: Supplementary file 1 — Supplementary Material 1 [file 12913_2024_11040_MOESM1_ESM.docx]

**Interview Guide**

**Introduction**

1. As an introduction, can you share a little about yourself and the work you do?
2. Can you describe the anaesthesia care situation in [country] in general?

**Ketamine in General**

1. I explained before that in this study we would like to get to know more about the use of ketamine as an anaesthetic in Sub-Saharan Africa. Can you describe in what way and to what extent ketamine is used in [country] for anaesthesia and surgical care?
2. Can you describe ketamine availability and use for anaesthesia in relation to other anaesthetics and their availability and use in [country]?
3. What would you say the importance is of ketamine for anaesthesia in [country]?
   1. If it is important: why is ketamine an important anaesthetic in [country]?

**Barriers to Anaesthesia Care**

1. You already described the anaesthesia care situation, but what do you think the main barriers to anaesthesia care are in [country]?
2. Does ketamine play a role in alleviating some of these barriers?
   1. If yes, how?

**Rwanda Case Study**

1. In a study we recently conducted in Rwanda, we looked at the availability of anaesthesia commodities at hospital level and higher. Availability of general anaesthetics was:

|  | **Availability** | |
| --- | --- | --- |
|  | N | % |
| Ketamine | 42 | 77.8 |
| Thiopental | 24 | 44.4 |
| Inhalant agents (halothane, isoflurane, sevoflurane) | 29 | 53.7 |
| Propofol | 44 | 81.5 |

How do these findings compare to the situation in your country? (Is Rwanda different compared to other Sub-Saharan African countries?)

**International Scheduling**

1. Are you familiar with the three international drug control conventions, which together establish internationally applicable control measures on narcotics and psychotropic substances (such as opioids, benzodiazepines, antipsychotics, etc.)? Their aim is to ensure the availability of these medicines for medical and scientific purposes, while at the same time preventing them from being diverted into illegal channels.

*(If yes, continue asking the question)*

*(If no, explain about the conventions)*

1. Ketamine is at the moment not internationally scheduled as a controlled substance. However, due to misuse in some high-income countries, especially in East and Southeast Asian countries like China, Hong Kong, Taiwan, and Japan, multiple attempts have been made to make ketamine an internationally controlled substance as well. What are your thoughts on the discussion at the international level about regulating ketamine more strictly?
2. In your opinion, do you think having ketamine scheduled internationally as a controlled substance, with subsequently the same types of measures put in place that regulate medicines such as morphine, oxycodone or fentanyl, would have an impact on its availability and use in [country]?

- *(Probe for more insights if necessary)*

1. Are there any special regulations in place for the manufacturing, distributing, use and handling of ketamine in [country]?

**Misuse of ketamine**

1. Are you aware of any incidences of misuse of ketamine in [country]?
2. Do you think ketamine misuse is a problem in your [country]?
3. How do you think misuse of ketamine can be prevented while at the same time ensuring it remains accessible for medical use?
4. Do you have any suggestions on how in the future access to anaesthesia care can be safeguarded or improved in [country], both with regards to ketamine but also looking broader than that?
5. Is there anything else you would like to share with me before we conclude?
