# Supplementary material for: The impact of scheduling ketamine as an internationally controlled substance on anaesthesia care in Sub-Saharan Africa: a case study and key informant interviews
Source: BMC Health Serv Res. 2024 May 7;24:598. doi: 10.1186/s12913-024-11040-w (PMC11077710; doi:10.1186/s12913-024-11040-w)
Supplement: Supplementary file 3 — Supplementary Material 3 [file 12913_2024_11040_MOESM3_ESM.docx]

**
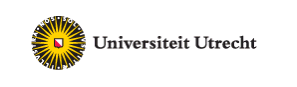

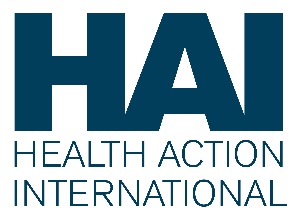
INFORMED CONSENT FORM**

**The impact of scheduling ketamine as an internationally controlled substance on surgical and anaesthesia care in Sub-Saharan Africa**

**Principal Researcher Organisation**

Gaby Ooms Health Action International

[gaby@haiweb.org](mailto:gaby@haiweb.org) Overtoom 60-2, 8252 GS

Amsterdam, The Netherlands

**Study background**

Access to surgical and anaesthesia care is a major issue for many people living in Sub-Saharan Africa, where more than 95% of the population does not have adequate access to it. In this region alone, an additional 41 million surgical procedures are needed each year. Lack of access to surgical and anaesthesia care in Sub-Saharan Africa is caused by lack of specialised healthcare workers, lack of basic infrastructure, lack of surgical and anaesthesia equipment, and lack of essential medicines. As a consequence, in much of Sub-Saharan Africa, surgical procedures often take place without anaesthesia or pain management. To alleviate the suffering of patients in these settings, hospitals have become reliant on ketamine for anaesthesia. Ketamine is listed as an anaesthetic on the WHO Model List of Essential Medicines.

Unfortunately, ketamine is misused in high-income countries, and especially in East and Southeast Asia, like China, Hong Kong, Taiwan, and Japan. Because of the misuse in these countries, China has submitted multiple requests to schedule ketamine internationally as a controlled substance, which would lead to more stringent regulation. The requests were denied, but it is likely that more will be made in the future. This research therefore aims to estimate the importance of ketamine for anaesthesia and surgical care in Sub-Saharan Africa, and what the impact might be of international scheduling of ketamine on access. This will be done through key informant interviews.

**Research team**

The principal investigator of this study is Gaby Ooms (HAI/Utrecht University, the Netherlands), who is supported by Dr. Mohammed Usman (Federal Medical Centre Birnin Kudu, Nigeria), Dr. Rianne van den Ham (Utrecht University, the Netherlands), Dr. Aukje Mantel-Teeuwisse (Utrecht University, the Netherlands), and Dr. Tim Reed (HAI, the Netherlands). Should you have any questions about the project, please feel free to contact the researchers.

**Data Collection**

This research project consists of key informant interviews. We would like to ask you to participate in an interview to get your insights on the relevance of ketamine for surgical and anaesthesia care in the country/countries where you work. The interview is expected to take about 30 minutes of your time. The interview is semi-structured and will be guided by a set of questions. It will be completed online (Zoom, Teams) or over telephone, according to your preference. With your permission, the interview will be recorded and transcribed for analysis purposes afterwards.

**Discomforts/risks**

The research team does not believe there are any foreseeable risks associated with this project. If you have any concern on this matter, we do encourage you to get in touch with us via the contact information at the end of this letter.

**Benefits**

Research is fundamental in healthcare. This project attempts to estimate the importance of ketamine for anaesthesia and surgical care in Sub-Saharan Africa, and what the impact will be of international scheduling of ketamine on access, so fitting action may be taken if necessary.

**Compensation**

No compensations can be granted for your participation in the study.

**Participant’s rights**

Participation in this study is completely voluntary. You are not under any obligation to participate. If you agree to participate, you can withdraw from the study at any time without consequences. Any data collected from you will be deleted and will not be used in the research. If you agree to take part, you are asked for your consent at the start of the interview. You will be provided with a copy of the information sheet and the consent form for your records.

**Questions**

Any time during or directly before or after the interview, the participant can ask the researchers questions related to the research. If the participant has questions after the research, the researchers can be reached by the email address as written on the first page of this participant information sheet.

**Confidentiality**

Information gathered about you will be held in strict confidence. Your name and any other identifying information will be removed from any data. A key (E.g., P1, P2, P3, etc.) will be used to refer to participants within the research. No individual respondents will be able to be identified in any publications or presentations. All data will be stored on a secure Drive and will only be made available to the researchers. The recordings of the interviews will be deleted after transcription is completed. The transcripts will be stored securely for a period of ten years, after which they will also be deleted.

**Study findings**

The findings of the research project will be published in a scientific peer-reviewed journal and may be presented at meetings and conferences. You can choose to receive a copy of the publication when the study is published. Should you wish to, then please indicate so in the interview.

**Complaints or concerns**

If you wish to make a complaint regarding the manner in which this research project is conducted, it should be directed to Tim Reed ([tim@haiweb.org](mailto:tim@haiweb.org), +31 (0) 20 412 4523). If you have any worries or complaints about your privacy, please contact [privacy-beta@uu.nl](mailto:privacy-beta@uu.nl). Any complaint or concern will be treated in confidence and will be fully investigated. You will be informed about the outcome.

The interviewer has discussed this information with me and offered to answer my questions. For any further questions, I may contact Gaby Ooms.

**STATEMENT OF CONSENT**

This is the statement you will be asked to consent to at the start of the interview.

*........................................................................... has described to me what is going to be done, the risks, the benefits involved and my rights regarding this study. I understand that my decision to participate in this study will not negatively affect me. In the use of the study information, my identity will be concealed. I am aware that I may withdraw at any time. I understand that by consenting to this statement, I do not waive any of my legal rights but merely indicate that I have been informed about the research study in which I am voluntarily agreeing to participate. A copy of this form will be provided to me.*

*Do you consent to participate in this research?*

**I hereby declare that I have read the information letter about the “impact of scheduling ketamine as an internationally controlled substance on surgical and anaesthesia care in Sub-Saharan Africa” study and agree to participate in the study.**

**Name Signature**

**Date**
